# 5-Bromo-2-methyl-3-(3-methyl­phenyl­sulfin­yl)-1-benzo­furan

**DOI:** 10.1107/S1600536814003365

**Published:** 2014-02-19

**Authors:** Hong Dae Choi, Pil Ja Seo, Uk Lee

**Affiliations:** a Department of Chemistry, Dongeui University, San 24 Kaya-dong, Busanjin-gu, Busan 614-714, Republic of Korea; b Department of Chemistry, Pukyong National University, 599-1 Daeyeon 3-dong, Nam-gu, Busan 608-737, Republic of Korea

## Abstract

In the title compound, C_16_H_13_BrO_2_S, the dihedral angle between the mean plane [r.m.s. deviation = 0.012 (1) Å] of the benzo­furan ring system and the 3-methyl­phenyl ring is 84.83 (4)°. In the crystal, mol­ecules are linked *via* pairs of Br⋯O [3.240 (1) Å] contacts, forming inversion dimers. These dimers are linked by C—H⋯π inter­actions, forming a three-dimensional network.

## Related literature   

For background information and the crystal structures of related compounds, see: Choi *et al.* (2010 ▶, 2012**a* ▶,b* ▶). For a review of halogen bonding, see: Politzer *et al.* (2007 ▶).
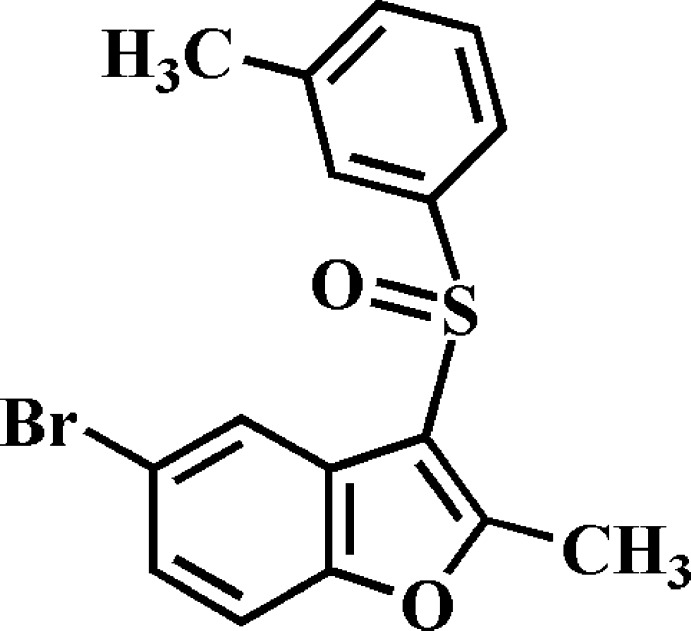


## Experimental   

### 

#### Crystal data  

C_16_H_13_BrO_2_S
*M* *_r_* = 349.23
Triclinic, 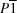

*a* = 8.4209 (1) Å
*b* = 8.9042 (1) Å
*c* = 10.8628 (1) Å
α = 106.956 (1)°
β = 90.441 (1)°
γ = 108.900 (1)°
*V* = 732.38 (1) Å^3^
*Z* = 2
Mo *K*α radiation
μ = 2.95 mm^−1^
*T* = 173 K
0.43 × 0.32 × 0.09 mm

#### Data collection  

Bruker SMART APEXII CCD diffractometer
Absorption correction: multi-scan (*SADABS*; Bruker, 2009 ▶) *T*_min_ = 0.365, *T*_max_ = 0.746
13736 measured reflections
3667 independent reflections
3391 reflections with *I* > 2σ(*I*)
*R*_int_ = 0.034

#### Refinement  

*R*[*F* ^2^ > 2σ(*F* ^2^)] = 0.025
*wR*(*F* ^2^) = 0.066
*S* = 1.09
3667 reflections
183 parameters
H-atom parameters constrained
Δρ_max_ = 0.36 e Å^−3^
Δρ_min_ = −0.54 e Å^−3^

### 

Data collection: *APEX2* (Bruker, 2009 ▶); cell refinement: *SAINT* (Bruker, 2009 ▶); data reduction: *SAINT*; program(s) used to solve structure: *SHELXS97* (Sheldrick, 2008 ▶); program(s) used to refine structure: *SHELXL97* (Sheldrick, 2008 ▶); molecular graphics: *ORTEP-3 for Windows* (Farrugia, 2012 ▶) and *DIAMOND* (Brandenburg, 1998 ▶); software used to prepare material for publication: *SHELXL97*.

## Supplementary Material

Crystal structure: contains datablock(s) I. DOI: 10.1107/S1600536814003365/su2700sup1.cif

Structure factors: contains datablock(s) I. DOI: 10.1107/S1600536814003365/su2700Isup2.hkl

Supporting information file. DOI: 10.1107/S1600536814003365/su2700Isup3.cml

CCDC reference:

Additional supporting information:  crystallographic information; 3D view; checkCIF report

## Figures and Tables

**Table 1:** Hydrogen-bond geometry (Å, °) *Cg*1 and *Cg*2 are the centroids of rings C10–C15 (3-methyl­phen­yl) and C2–C7 (benzene), respectively.

*D*—H⋯*A*	*D*—H	H⋯*A*	*D*⋯*A*	*D*—H⋯*A*
C9—H9*A*⋯*Cg*1^i^	0.98	2.91	3.785 (2)	150
C12—H12⋯*Cg*2^ii^	0.95	2.79	3.674 (2)	154

Symmetry codes: (i) 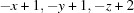; (ii) 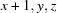.

**Fig. 1.**
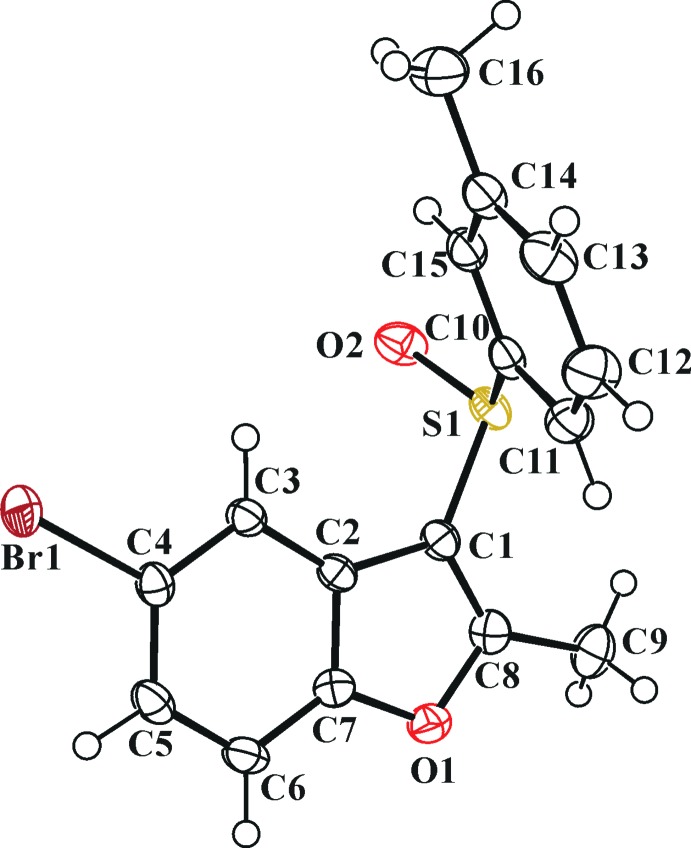
The molecular structure of the title molecule, with atom labelling. Displacement ellipsoids are drawn at the 50% probability level.

**Fig. 2.**
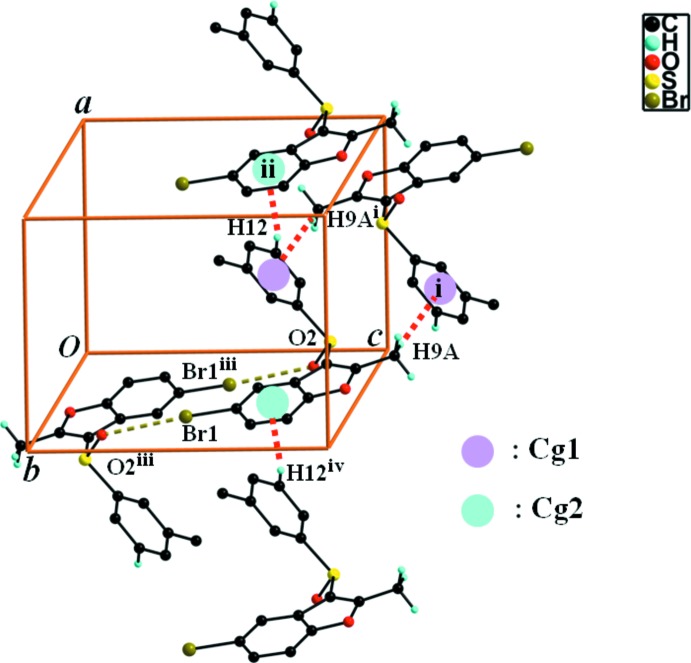
A view along the b axis of the crystal packing of the title compound, showing the C—H···π and Br···O interactions as dotted lines [see Table 1 for details; H atoms not involved in hydrogen-bonding have been omitted for clarity; symmetry codes : (i) - x + 1, - y + 1, - z + 2; (ii) x + 1, y, z; (iii) - x, - y + 1, - z + 1; (iv) x - 1, y, z].

## supplementary crystallographic information

### 1. Comment

As a part of our continuing study of 5-bromo-2-methyl-1-benzofuran derivatives containing 4-chlorophenylsulfinyl (Choi *et al.*, 2010), 4-methylphenylsulfinyl (Choi *et al.*, 2012*a*) and 3-fluorophenylsulfinyl (Choi *et al.*, 2012*b*) substituents in the 3-position, we report herein on the crystal structure of the title compound.

In the title molecule, Fig. 1, the benzofuran unit is essentially planar, with a mean deviation of 0.012 (1) Å from the mean plane defined by the nine constituent atoms. It is inclined to the 3-methylphenyl ring by 84.83 (4)°.

In the crystal, Fig. 2, molecules are connected by pairs of Br···O halogen-bonds (Politzer *et al.*, 2007), between the bromine atom and the O atom of the S═O unit [Br1···O2^iii^ = 3.240 (1) Å, C4—Br1···O2^iii^ = 175.63 (5)°; symmetry code: (iii) - *x*, - *y* + 1, - *z* + 1] forming inversion dimers. These dimers are linked by C—H···π interactions into supramolecular chains running along the *a*-axis. In addition, there are C—H···π interactions resulting in inversion related dimers (Table 1 and Fig. 2).

### 2. Experimental

3-Chloroperoxybenzoic acid (77%, 224 mg, 1.0 mmol) was added in small portions to a stirred solution of 5-bromo-2-methyl-3-(3-methylphenylsulfanyl)-1-benzofuran (300 mg, 0.9 mmol) in dichloromethane (30 mL) at 273 K. After being stirred at room temperature for 5h, the mixture was washed with saturated sodium bicarbonate solution and the organic layer was separated, dried over magnesium sulfate, filtered and concentrated at reduced pressure. The residue was purified by column chromatography (hexane–ethyl acetate, 2:1 v/v) to afford the title compound as a colorless solid [Yield 78%, M.p. 413–414 K; *R*_f_ = 0.49 (hexane-ethyl acetate, 2:1 v/v)]. Single crystals suitable for X-ray diffraction were prepared by slow evaporation of a solution of the title compound in ethyl acetate at room temperature.

### 3. Refinement

All the H atoms were positioned geometrically and refined using a riding model: C—H = 0.95 Å for aryl and 0.99 Å for methyl H atoms with *U*_iso_(H) = 1.2*U*_eq_(C-aryl) and = 1.5*U*_eq_(C-methyl). The positions of the methyl hydrogens were optimized rotationally.

### Crystal data

**Table:**

C_16_H_13_BrO_2_S	*Z* = 2
*M**_r_* = 349.23	*F*(000) = 352
Triclinic, *P*1	*D*_x_ = 1.584 Mg m^−^^3^
Hall symbol: -P 1	Mo *K*α radiation, λ = 0.71073 Å
*a* = 8.4209 (1) Å	Cell parameters from 8553 reflections
*b* = 8.9042 (1) Å	θ = 2.5–28.4°
*c* = 10.8628 (1) Å	µ = 2.95 mm^−^^1^
α = 106.956 (1)°	*T* = 173 K
β = 90.441 (1)°	Block, colourless
γ = 108.900 (1)°	0.43 × 0.32 × 0.09 mm
*V* = 732.38 (1) Å^3^	

### Data collection

**Table:**

Bruker SMART APEXII CCD diffractometer	3667 independent reflections
Radiation source: rotating anode	3391 reflections with *I* > 2σ(*I*)
Graphite multilayer monochromator	*R*_int_ = 0.034
Detector resolution: 10.0 pixels mm^-1^	θ_max_ = 28.4°, θ_min_ = 2.0°
φ and ω scans	*h* = −11→11
Absorption correction: multi-scan (*SADABS*; Bruker, 2009)	*k* = −11→11
*T*_min_ = 0.365, *T*_max_ = 0.746	*l* = −14→14
13736 measured reflections	

### Refinement

**Table:**

Refinement on *F*^2^	Primary atom site location: structure-invariant direct methods
Least-squares matrix: full	Secondary atom site location: difference Fourier map
*R*[*F*^2^ > 2σ(*F*^2^)] = 0.025	Hydrogen site location: difference Fourier map
*wR*(*F*^2^) = 0.066	H-atom parameters constrained
*S* = 1.09	*w* = 1/[σ^2^(*F*_o_^2^) + (0.0306*P*)^2^ + 0.2396*P*] where *P* = (*F*_o_^2^ + 2*F*_c_^2^)/3
3667 reflections	(Δ/σ)_max_ = 0.001
183 parameters	Δρ_max_ = 0.36 e Å^−^^3^
0 restraints	Δρ_min_ = −0.54 e Å^−^^3^

### Special details

**Table:**

Geometry. All esds (except the esd in the dihedral angle between two l.s. planes) are estimated using the full covariance matrix. The cell esds are taken into account individually in the estimation of esds in distances, angles and torsion angles; correlations between esds in cell parameters are only used when they are defined by crystal symmetry. An approximate (isotropic) treatment of cell esds is used for estimating esds involving l.s. planes.
Refinement. Refinement of F^2^ against ALL reflections. The weighted R-factor wR and goodness of fit S are based on F^2^, conventional R-factors R are based on F, with F set to zero for negative F^2^. The threshold expression of F^2^ > 2sigma(F^2^) is used only for calculating R-factors(gt) etc. and is not relevant to the choice of reflections for refinement. R-factors based on F^2^ are statistically about twice as large as those based on F, and R- factors based on ALL data will be even larger.

### Fractional atomic coordinates and isotropic or equivalent isotropic displacement parameters (Å^2^)

**Table:**

	*x*	*y*	*z*	*U*_iso_*/*U*_eq_	
Br1	0.04187 (2)	0.75979 (2)	0.479648 (15)	0.03138 (7)	
S1	0.24413 (5)	0.49344 (5)	0.90916 (4)	0.02597 (9)	
O1	0.23301 (15)	0.94970 (14)	1.04896 (11)	0.0283 (2)	
O2	0.08675 (15)	0.36872 (15)	0.82700 (13)	0.0339 (3)	
C1	0.23351 (19)	0.69433 (19)	0.93705 (15)	0.0230 (3)	
C2	0.18550 (18)	0.76541 (18)	0.84534 (14)	0.0209 (3)	
C3	0.14284 (19)	0.71368 (19)	0.71172 (15)	0.0224 (3)	
H3	0.1423	0.6081	0.6579	0.027*	
C4	0.10115 (19)	0.82404 (19)	0.66114 (15)	0.0235 (3)	
C5	0.0980 (2)	0.9788 (2)	0.73792 (17)	0.0271 (3)	
H5	0.0661	1.0488	0.6989	0.033*	
C6	0.1413 (2)	1.03082 (19)	0.87084 (17)	0.0273 (3)	
H6	0.1405	1.1358	0.9249	0.033*	
C7	0.18532 (19)	0.92194 (19)	0.92009 (15)	0.0234 (3)	
C8	0.2602 (2)	0.8082 (2)	1.05582 (16)	0.0266 (3)	
C9	0.3152 (3)	0.8114 (3)	1.18646 (17)	0.0384 (4)	
H9A	0.3161	0.7005	1.1833	0.058*	
H9B	0.2369	0.8424	1.2469	0.058*	
H9C	0.4291	0.8934	1.2159	0.058*	
C10	0.40882 (18)	0.50755 (19)	0.80452 (15)	0.0234 (3)	
C11	0.5604 (2)	0.6403 (2)	0.84122 (18)	0.0317 (3)	
H11	0.5760	0.7300	0.9187	0.038*	
C12	0.6883 (2)	0.6386 (2)	0.7623 (2)	0.0384 (4)	
H12	0.7932	0.7281	0.7859	0.046*	
C13	0.6648 (2)	0.5077 (2)	0.64917 (19)	0.0345 (4)	
H13	0.7539	0.5090	0.5959	0.041*	
C14	0.5133 (2)	0.3745 (2)	0.61235 (17)	0.0289 (3)	
C15	0.3854 (2)	0.3760 (2)	0.69235 (16)	0.0266 (3)	
H15	0.2810	0.2857	0.6697	0.032*	
C16	0.4876 (3)	0.2305 (3)	0.4904 (2)	0.0424 (4)	
H16A	0.5240	0.2737	0.4181	0.064*	
H16B	0.3678	0.1614	0.4714	0.064*	
H16C	0.5545	0.1629	0.5023	0.064*	

### Atomic displacement parameters (Å^2^)

**Table:**

	*U*^11^	*U*^22^	*U*^33^	*U*^12^	*U*^13^	*U*^23^
Br1	0.03812 (10)	0.03427 (10)	0.02629 (9)	0.01357 (7)	0.00008 (6)	0.01480 (7)
S1	0.02985 (19)	0.02419 (19)	0.0310 (2)	0.01154 (15)	0.00610 (15)	0.01633 (16)
O1	0.0335 (6)	0.0250 (6)	0.0242 (6)	0.0104 (5)	0.0017 (4)	0.0042 (5)
O2	0.0277 (6)	0.0229 (6)	0.0491 (8)	0.0053 (5)	0.0083 (5)	0.0119 (5)
C1	0.0248 (7)	0.0231 (7)	0.0238 (7)	0.0085 (6)	0.0040 (5)	0.0107 (6)
C2	0.0214 (6)	0.0196 (7)	0.0245 (7)	0.0077 (5)	0.0052 (5)	0.0099 (6)
C3	0.0259 (7)	0.0199 (7)	0.0233 (7)	0.0092 (5)	0.0032 (5)	0.0079 (6)
C4	0.0225 (7)	0.0249 (7)	0.0256 (7)	0.0075 (6)	0.0022 (5)	0.0121 (6)
C5	0.0260 (7)	0.0227 (7)	0.0373 (9)	0.0088 (6)	0.0034 (6)	0.0156 (7)
C6	0.0292 (8)	0.0184 (7)	0.0345 (8)	0.0093 (6)	0.0036 (6)	0.0070 (6)
C7	0.0241 (7)	0.0212 (7)	0.0237 (7)	0.0069 (5)	0.0031 (5)	0.0063 (6)
C8	0.0259 (7)	0.0286 (8)	0.0264 (8)	0.0080 (6)	0.0039 (6)	0.0113 (6)
C9	0.0439 (10)	0.0467 (11)	0.0243 (8)	0.0141 (8)	−0.0007 (7)	0.0124 (8)
C10	0.0219 (7)	0.0236 (7)	0.0296 (8)	0.0100 (6)	0.0012 (6)	0.0131 (6)
C11	0.0277 (8)	0.0259 (8)	0.0368 (9)	0.0069 (6)	0.0001 (7)	0.0057 (7)
C12	0.0253 (8)	0.0308 (9)	0.0524 (12)	0.0037 (7)	0.0050 (7)	0.0097 (8)
C13	0.0293 (8)	0.0335 (9)	0.0450 (10)	0.0131 (7)	0.0123 (7)	0.0157 (8)
C14	0.0315 (8)	0.0285 (8)	0.0316 (8)	0.0152 (6)	0.0026 (6)	0.0111 (7)
C15	0.0245 (7)	0.0225 (7)	0.0338 (8)	0.0081 (6)	−0.0015 (6)	0.0103 (6)
C16	0.0451 (10)	0.0391 (10)	0.0401 (10)	0.0190 (8)	0.0048 (8)	0.0027 (8)

### Geometric parameters (Å, º)

**Table:**

Br1—C4	1.8988 (16)	C8—C9	1.479 (2)
Br1—O2^i^	3.2399 (14)	C9—H9A	0.9800
S1—O2	1.4954 (13)	C9—H9B	0.9800
S1—C1	1.7571 (16)	C9—H9C	0.9800
S1—C10	1.7960 (16)	C10—C15	1.382 (2)
O1—C8	1.373 (2)	C10—C11	1.387 (2)
O1—C7	1.3810 (19)	C11—C12	1.382 (3)
C1—C8	1.352 (2)	C11—H11	0.9500
C1—C2	1.444 (2)	C12—C13	1.386 (3)
C2—C3	1.394 (2)	C12—H12	0.9500
C2—C7	1.394 (2)	C13—C14	1.389 (2)
C3—C4	1.388 (2)	C13—H13	0.9500
C3—H3	0.9500	C14—C15	1.390 (2)
C4—C5	1.396 (2)	C14—C16	1.507 (3)
C5—C6	1.387 (2)	C15—H15	0.9500
C5—H5	0.9500	C16—H16A	0.9800
C6—C7	1.377 (2)	C16—H16B	0.9800
C6—H6	0.9500	C16—H16C	0.9800
			
C4—Br1—O2^i^	175.63 (5)	C8—C9—H9B	109.5
O2—S1—C1	108.56 (7)	H9A—C9—H9B	109.5
O2—S1—C10	106.16 (7)	C8—C9—H9C	109.5
C1—S1—C10	98.49 (7)	H9A—C9—H9C	109.5
C8—O1—C7	106.43 (12)	H9B—C9—H9C	109.5
C8—C1—C2	107.45 (14)	C15—C10—C11	121.21 (15)
C8—C1—S1	123.80 (12)	C15—C10—S1	118.01 (12)
C2—C1—S1	128.62 (12)	C11—C10—S1	120.56 (13)
C3—C2—C7	119.50 (13)	C12—C11—C10	118.38 (17)
C3—C2—C1	135.70 (14)	C12—C11—H11	120.8
C7—C2—C1	104.80 (13)	C10—C11—H11	120.8
C4—C3—C2	116.72 (14)	C11—C12—C13	120.62 (16)
C4—C3—H3	121.6	C11—C12—H12	119.7
C2—C3—H3	121.6	C13—C12—H12	119.7
C3—C4—C5	122.96 (15)	C12—C13—C14	121.11 (17)
C3—C4—Br1	118.47 (12)	C12—C13—H13	119.4
C5—C4—Br1	118.57 (11)	C14—C13—H13	119.4
C6—C5—C4	120.40 (14)	C13—C14—C15	118.14 (16)
C6—C5—H5	119.8	C13—C14—C16	121.43 (17)
C4—C5—H5	119.8	C15—C14—C16	120.43 (16)
C7—C6—C5	116.32 (14)	C10—C15—C14	120.54 (15)
C7—C6—H6	121.8	C10—C15—H15	119.7
C5—C6—H6	121.8	C14—C15—H15	119.7
C6—C7—O1	125.57 (14)	C14—C16—H16A	109.5
C6—C7—C2	124.08 (15)	C14—C16—H16B	109.5
O1—C7—C2	110.35 (13)	H16A—C16—H16B	109.5
C1—C8—O1	110.95 (14)	C14—C16—H16C	109.5
C1—C8—C9	133.31 (16)	H16A—C16—H16C	109.5
O1—C8—C9	115.71 (15)	H16B—C16—H16C	109.5
C8—C9—H9A	109.5		
			
O2—S1—C1—C8	129.11 (14)	C1—C2—C7—O1	1.19 (16)
C10—S1—C1—C8	−120.59 (14)	C2—C1—C8—O1	0.05 (18)
O2—S1—C1—C2	−46.22 (16)	S1—C1—C8—O1	−176.13 (11)
C10—S1—C1—C2	64.09 (15)	C2—C1—C8—C9	−178.18 (18)
C8—C1—C2—C3	179.77 (17)	S1—C1—C8—C9	5.6 (3)
S1—C1—C2—C3	−4.3 (3)	C7—O1—C8—C1	0.68 (17)
C8—C1—C2—C7	−0.75 (17)	C7—O1—C8—C9	179.25 (14)
S1—C1—C2—C7	175.18 (12)	O2—S1—C10—C15	−21.27 (13)
C7—C2—C3—C4	−0.4 (2)	C1—S1—C10—C15	−133.50 (12)
C1—C2—C3—C4	179.07 (16)	O2—S1—C10—C11	164.08 (13)
C2—C3—C4—C5	−1.1 (2)	C1—S1—C10—C11	51.85 (14)
C2—C3—C4—Br1	−179.96 (11)	C15—C10—C11—C12	0.3 (3)
C3—C4—C5—C6	1.5 (2)	S1—C10—C11—C12	174.82 (14)
Br1—C4—C5—C6	−179.67 (12)	C10—C11—C12—C13	0.2 (3)
C4—C5—C6—C7	−0.3 (2)	C11—C12—C13—C14	−0.4 (3)
C5—C6—C7—O1	179.71 (14)	C12—C13—C14—C15	−0.1 (3)
C5—C6—C7—C2	−1.2 (2)	C12—C13—C14—C16	−179.16 (18)
C8—O1—C7—C6	178.02 (15)	C11—C10—C15—C14	−0.8 (2)
C8—O1—C7—C2	−1.18 (17)	S1—C10—C15—C14	−175.41 (12)
C3—C2—C7—C6	1.6 (2)	C13—C14—C15—C10	0.6 (2)
C1—C2—C7—C6	−178.02 (15)	C16—C14—C15—C10	179.75 (15)
C3—C2—C7—O1	−179.22 (13)		

Symmetry code: (i) −*x*, −*y*+1, −*z*+1.

### Hydrogen-bond geometry (Å, º)

Cg1 and Cg2 are the centroids of rings C10–C15 (3-methylphenyl) and C2–C7 (benzene), respectively.

**Table:**

*D*—H···*A*	*D*—H	H···*A*	*D*···*A*	*D*—H···*A*
C9—H9*A*···*Cg*1^ii^	0.98	2.91	3.785 (2)	150
C12—H12···*Cg*2^iii^	0.95	2.79	3.674 (2)	154

Symmetry codes: (ii) −*x*+1, −*y*+1, −*z*+2; (iii) *x*+1, *y*, *z*.
